# The Effects of Restrictive Fluid Resuscitation on the Clinical Outcomes in Patients with Sepsis or Septic Shock: A Meta-Analysis of Randomized-Controlled Trials

**DOI:** 10.7759/cureus.45620

**Published:** 2023-09-20

**Authors:** Husna Shahnoor, Rachana Divi, Lokeshwar Raaju Addi Palle, Ashutosh Sharma, Bianca Contractor, Santoshi Krupanagaram, Saima Batool, Neelum Ali

**Affiliations:** 1 Internal Medicine, Deccan College of Medical Sciences, Hyderabad, IND; 2 Medicine and Surgery, GSL Medical College, Hyderabad, IND; 3 Surgery, Kamala Children's Hospital, Chennai, IND; 4 Medicine, Kathmandu Medical College and Teaching Hospital, Kathmandu, NPL; 5 Internal Medicine, Smt. NHL Municipal Medical College, Ahmedabad, IND; 6 Medicine, MNR Medical College and Hospital, Sangareddy, IND; 7 Internal Medicine, Hameed Latif Hospital, Lahore, PAK; 8 Internal Medicine, University of Health Sciences, Lahore, PAK

**Keywords:** meta-analysis, septic shock, sepsis, resuscitation, fluid restriction

## Abstract

This study aims to assess the impact of a restrictive resuscitation strategy on the outcomes of patients with sepsis and septic shock. This meta-analysis was conducted in accordance with the recommendations from the Preferred Reporting Items for Systematic Reviews and Meta-Analysis Protocols (PRISMA-P) guidelines. A systematic search was performed in databases, including PubMed, Web of Science, EMBASE, and the Cochrane Library, covering the period from the inception of the database to August 2023, with no limitations on the language of publication. Outcomes assessed in the meta-analysis included mortality, duration of intensive care unit (ICU) stay in days, duration of mechanical ventilation in days, acute kidney injury (AKI) or the need for renal replacement therapy (RRT), and length of hospital stay in days. Overall, 12 studies met the inclusion criteria and were included in the present meta-analysis. The findings of this study indicate that although the risk of mortality was lower in fluid restriction compared to the control group, the difference was statistically insignificant (risk ratio (RR): 0.98; 95% confidence interval (CI): 0.9-1.05; P value: 0.61). Additionally, the duration of mechanical ventilation was significantly shorter in the restrictive fluid group compared to its counterparts (mean difference (MD): -1.02; 95% CI: -1.65 to -0.38; P value: 0.003). There were no significant differences found in relation to the duration of ICU stays, the incidence of AKI, the requirement for RRT, or the length of hospital stays measured in days.

## Introduction and background

Sepsis poses a significant global health challenge, with approximately 49 million new cases and 11 million associated deaths reported annually [1]. Sepsis is characterized by life-threatening organ dysfunction resulting from an uncontrolled response to infection, while septic shock is an advanced stage of sepsis characterized by severe circulatory dysfunction, a notably sharp drop in blood pressure, which can ultimately lead to organ dysfunction and failure due to inadequate blood flow and oxygen delivery, carrying a higher risk of mortality compared to sepsis alone [2]. The primary approach to treating sepsis in its initial stages involves administering intravenous antibiotics and fluids, controlling the source of infection, and providing necessary supportive care [3]. In the context of septic shock, the foremost component of hemodynamic support is fluid administration, with a crucial focus on optimizing preload. However, recent observations have raised concerns about the potential harm of overly aggressive fluid resuscitation, as excessive positive fluid balance has been associated with increased mortality in intensive care units (ICUs) [4-5]. While various hemodynamic protocols have been studied in randomized controlled trials during the early hours of septic shock treatment [6], there is limited evidence concerning the practical aspects of fluid administration in the later stages.

Numerous physiological studies have highlighted the unreliability of static preload indices, such as central venous pressure (CVP), in assessing fluid responsiveness, especially in septic patients [7]. In contrast, dynamic preload indices like pulse pressure variation (PPV) or changes in stroke volume during passive leg raising (PLR) have proven to be highly dependable for evaluating fluid responsiveness, provided that the appropriate conditions for accurate PPV measurement are met [8].

Hospitals worldwide administer over 200 million liters of 0.9% sodium chloride intravenously every year [9]. Given this substantial volume of fluid administration, experts argue that each type of fluid possesses its own therapeutic index and recommend implementing active fluid restriction and deresuscitation strategies for septic patients after the initial resuscitation phase [9]. Pharmacists can play a crucial role in these strategies, from selecting and dosing fluids appropriately to overseeing pharmacist-driven deresuscitation protocols [10]. Despite the conflicting data surrounding fluid resuscitation in septic patients, there is an ongoing need for studies to determine the ideal volume and timing of fluid resuscitation, both in the initial resuscitation phase (within the first six hours) and beyond (during the restriction phase).

Although there is a consensus on the importance of adequate fluid therapy in sepsis, and despite numerous recent clinical trials exploring fluid management in sepsis, the optimal fluid management strategy remains contentious and unclear, lacking definitive guidelines for the ideal fluid resuscitation approach in critically ill septic patients. This study aims to assess the impact of a restrictive resuscitation strategy on outcomes in patients with sepsis and septic shock.

## Review

Methods

This meta-analysis was conducted in accordance with the recommendations from the Preferred Reporting Items for Systematic Reviews and Meta-Analysis Protocols (PRISMA-P guidelines) [11].

Search Strategy

A systematic search was performed in databases, including PubMed, Web of Science, EMBASE, and the Cochrane Library, covering the period from the inception of the database to August 2023, with no limitations on the language of publication. The keywords used for searching relevant articles included "restrictive resuscitation," "sepsis," and "standard care," along with their symptoms and Medical Subject Heading (MeSH) terms. Additionally, the reference lists of all included studies were manually screened to identify relevant studies.

Study Selection and Eligibility Criteria

We included studies that were randomized controlled trials (RCTs) comparing restrictive resuscitation with standard care or other resuscitation approaches. The study population comprised adult patients with sepsis or septic shock. We excluded observational studies, reviews, editorials, and expert opinions. Eligible records were imported into ENDNOTE software (X9 version). After removing duplicate studies, two reviewers independently conducted an initial assessment of the titles and abstracts. Articles meeting the criteria underwent a comprehensive full-text screening. In cases where there were differences in opinion between the two reviewers, resolution was achieved through discussion, consensus, or involving a third author. Subsequently, studies not relevant to the research criteria were excluded, with explicit reasons for their exclusion documented.

Data Extraction

Data from the included studies were extracted using a predesigned data collection table created in a Microsoft Excel Spreadsheet. Data extraction encompassed the following elements: author name, year of publication, sample size, participant characteristics, and outcomes. Outcomes assessed in the meta-analysis included mortality, duration of intensive care unit (ICU) stay in days, duration of mechanical ventilation in days, acute kidney injury (AKI) or the need for renal replacement therapy (RRT), and length of hospital stay in days.

Quality Assessment

Two reviewers independently assessed the potential bias in the included studies using the quality assessment from the Cochrane Collaboration. Seven aspects of bias were examined: (1) generation of random sequences (selection bias), (2) concealment of allocation (selection bias), (3) masking of participants and staff (performance bias), (4) blinding of outcome evaluation (detection bias), (5) incomplete outcome information (attrition bias), (6) selective reporting (reporting bias), and (7) other factors (follow-up duration, baseline characteristics).

Data Analysis

All data were analyzed using REVMAN (version 5.4.1) software to determine pooled effects. For continuous outcomes, the mean difference (MD) with a 95% confidence interval (CI) was calculated, and, for categorical outcomes, the risk ratio (RR) was reported with a 95% CI. A significance level of P < 0.05 was considered to indicate a significant difference. Heterogeneity among the study results was reported as I-square. I-square values of 0% to 25% showed low heterogeneiety, 25% to 50% represented moderate heterogeneity, 50% to 90% represented substantial heterogeneity, and 75% to 100% represented considerable heterogeneity.

Results

**Figure 1:**
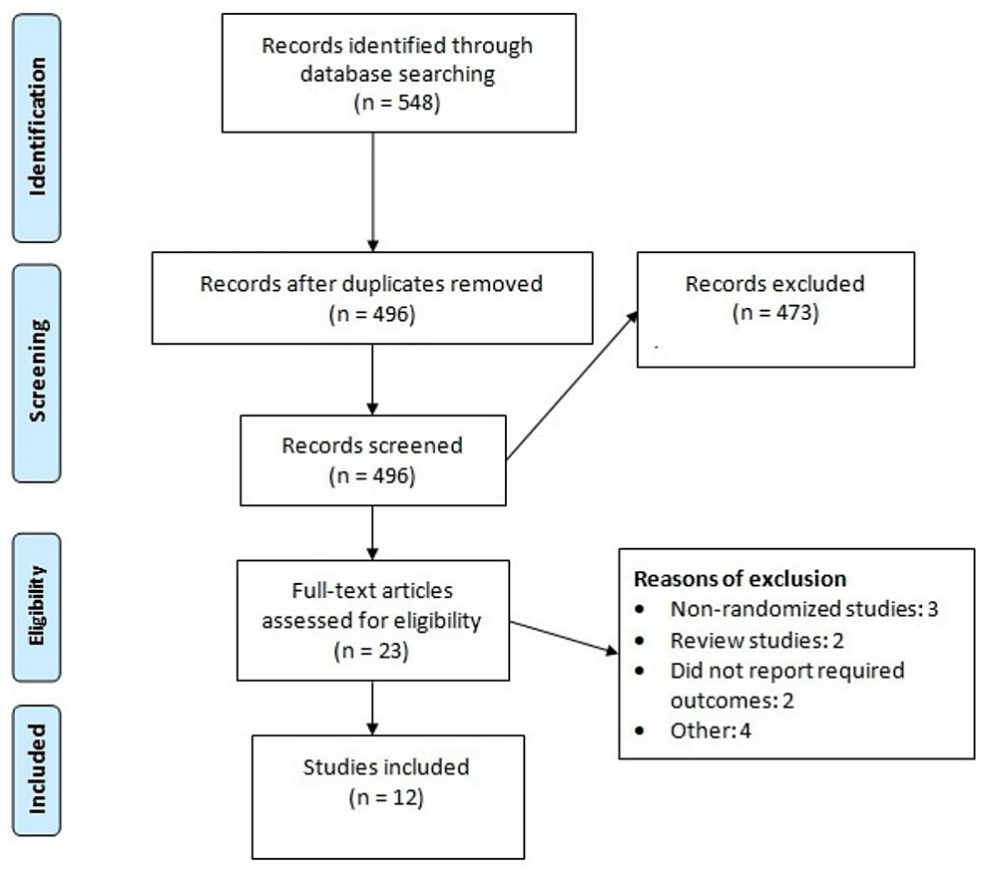
PRISMA-P flowchart showing the study selection process

**Figure 2:**
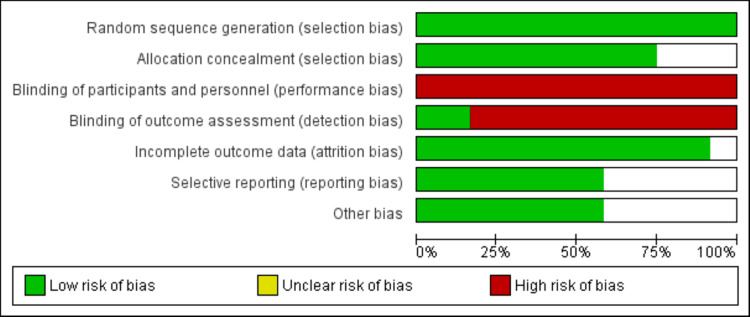
Quality assessment of included studies

After a comprehensive search, a total of 548 records were imported into ENDNOTE software. After removing 52 duplicates, the abstracts and titles of the remaining 406 studies were assessed. The full text of 23 studies was obtained and detailed assessment was done based on predefined inclusion and exclusion criteria. Through reading of the full text, 12 studies met the inclusion criteria and were included in the present meta-analysis. Figure 1 shows the PRISMA-P flowchart of study selection. Table 1 shows the characteristics of the included studies. Figure 2 shows the quality assessment of included RCTs.

**Table 1:** Characteristics of the included studies

Author Name	Year	Groups	Sample Size	Mean Age (Years)	Male (n)
Andrews et al. [12]	2017	Restrictive fluid	106	37.5	62
		Control	103	35.8	55
Chen et al. [13]	2015	Restrictive fluid	41	58	20
		Control	41	60	21
Corl et al. [14]	2015	Restrictive fluid	55	71	24
		Control	54	73.5	26
Douglas et al. [15]	2020	Restrictive fluid	83	61.8	32
		Control	41	62.7	28
Hjortrup et al. [16]	2016	Restrictive fluid	75	69	52
		Control	76	73	47
Jessen et al. [17]	2022	Restrictive fluid	61	75	37
		Control	62	76	34
Kjær et al. [18]	2023	Restrictive fluid	767	NR	NR
		Control	782		
Macdonald et al. [19]	2019	Restrictive fluid	50	66	31
		Control	49	66	30
Meyhoff et al. [20]	2021	Restrictive fluid	755	71	452
		Control	776	70	452
Noureldin et al. [21]	2023	Restrictive fluid	40	NR	NR
		Control	40		
Richard et al. [22]	2015	Restrictive fluid	30	65	21
		Control	30	64	22
Shapiro et al. [23]	2023	Restrictive fluid	782	59.1	411
		Control	781	59.9	415

Mortality

**Figure 3:**
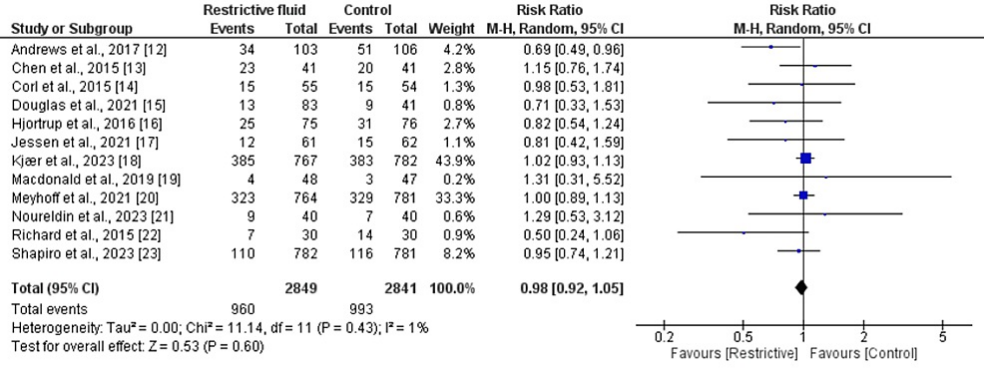
Comparison of all-cause mortality between groups Sources: References [12-23]

All 12 studies compared the mortality between restrictive resuscitation and standard care groups. As shown in Figure 3, the risk of mortality was higher in the standard care group compared to the restrictive restriction group, but the difference was statistically insignificant (RR: 0.98; 95% CI: 0.9 to 1.05; P value: 0.61). There is an insignificant heterogeneity.

Number of Days in Ventilation

**Figure 4:**
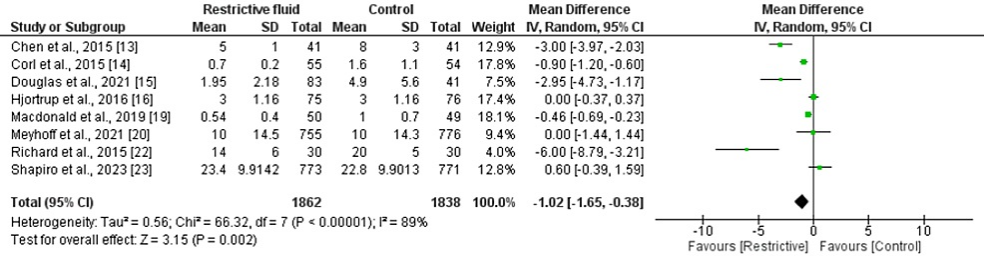
Comparison of the number of days in ventilation between two groups Sources: References [13-16, 19-20, 22-23]

Eight studies were included in the pooled analysis of the number of days in ventilation. As shown in Figure 4, the mean of ventilation days was significantly lower in the restrictive fluid group compared to the other group (MD: -1.02; 95% CI: -1.65 to -0.38; P value: 0.003). There is an insignificant heterogeneity.

Length of Stay in ICU

Six studies were included in the pooled analysis of the mean length of stay in the ICU. As shown in Table 2, no significant difference was found in the mean length of ICU stay between the two groups (MD: -0.04; 95% CI: -0.46 to 0.39; P value: 0.87). Significant heterogeneity was reported among the study results.

**Table 2:** Analysis of secondary outcomes between two groups RRT: renal replacement therapy; AKI: acute kidney injury; LOS: length of stay; ICU: intensive care unit; MD: mean difference; CI: confidence interval * Reported as risk ratio

Outcome	MD	95% CI	I-square
ICU stay (days)	-0.04	-0.46 to 0.39	88%
AKI or need of RRT	0.89*	0.77 to 1.03	9%
Hospital LOS	0.8	-0.50 to 2.11	85%

Acute kidney Injury (AKI) or Need for Renal Replacement Therapy (RRT)

Seven studies assessed the impact of restrictive fluid resuscitation on AKI or RRT. As shown in Table 2, the risk of AKI or RRT was higher in patients randomized in the restrictive fluid resuscitation group compared to the patients in the other group (RR: 0.89; 95% CI: 0.77 to 1.03; P value: 0.12). No significant heterogeneity was reported among the study results.

Hospital Length of Stay

Through a pooled analysis of four studies that assessed the duration of hospital stays, no significant difference was found in the mean of hospital duration of stay MD: 0.80 (with a 95% confidence interval ranging from -0.50 to 2.11) and a P value of 0.23. However, it is worth noting that there was substantial heterogeneity observed among the outcomes of these studies.

Discussion

This meta-analysis was conducted to assess the effect of fluid restriction on patients with sepsis and septic shock. The findings of this study indicate that although the risk of mortality was lower in fluid restriction compared to other approaches, the difference was statistically insignificant. Additionally, the duration of mechanical ventilation was significantly lower in the restrictive fluid group compared to its counterparts. The meta-analysis conducted by Reynolds et al. also comprised eight RCTs and reported similar findings [24].

Our study's discovery of a shorter period of mechanical ventilation when employing a restricted volume approach in septic patients aligns with previous research that has indicated the benefits of such an approach in reducing the duration of mechanical ventilation in cases of acute lung injury [25] and preventing excessive pulmonary fluid buildup in acute pancreatitis [26]. These findings not only support earlier systematic reviews that suggested a trend toward reduced mechanical ventilation duration but also strengthen the evidence base by incorporating new data that surpasses the threshold of statistical significance [24,27]. Significantly, our study did not identify any concerning signals related to adverse effects like acute kidney injury (AKI), digital ischemia, or increased vasopressor requirements associated with a restrictive resuscitation approach beyond the initial six-hour resuscitation phase. Consequently, it appears that implementing a restricted fluid strategy is not only safe but also justified after the initial six-hour resuscitation bundle, which is particularly relevant to our own research.

Pharmacists have the opportunity to play an active part in managing fluid levels and reducing excess fluids in critically ill patients. Research has indicated that a multidisciplinary approach, which emphasizes limiting fluid intake and promoting urine production during the initial 72 hours after recovering from shock, is linked to achieving a more balanced fluid state, increased days without needing intensive care, and a decrease in hospital mortality rates [10]. The findings from this meta-analysis add additional confirmation to the safety and potential advantages of these approaches and offer a foundation for further investigation into controlled fluid management for patients with sepsis.

This meta-analysis contributes to the existing body of research on the safety of fluid restriction. It does so by delving into previously identified safety indicators, including factors such as ICU stay, acute kidney injury (AKI), or the need for renal replacement therapy (RRT). The results showed that there were no statistically significant differences between the two groups in terms of any of these events.

It is also crucial for healthcare professionals to recognize that there is still uncertainty about the pros and cons of intravenous (IV) fluid therapy, especially during the initial stages of resuscitation. It is worth noting that a significant multicenter study exploring a strategy of limited fluid use in patients undergoing elective major abdominal surgery actually revealed poorer outcomes, despite smaller studies and observational data supporting this approach [28]. Currently, several large-scale randomized trials are underway to investigate various hemodynamic resuscitation protocols for patients in septic shock. These trials are expected to fill the current knowledge gap and provide valuable insights for both patients and healthcare providers.

The current meta-analysis has certain limitations. First, the extent of mortality outcome was primarily influenced by a single, larger RCT [18], highlighting the need for additional high-quality studies with minimal bias to generate more robust conclusions. Second, in all these studies, fluid restriction was implemented either during or after the six-hour resuscitation period (in which all studies administered at least 30 ml/kg of fluids within the initial six hours). Consequently, these studies were unable to evaluate the recommendation from the Surviving Sepsis Campaign, which advises administering 30 ml/kg of fluids within the initial three hours of fluid resuscitation for septic patients. One of the current trials compares restricted and liberal fluid in sepsis (NCT05453565), which will validate the findings of this meta-analysis. 

## Conclusions

In conclusion, this meta-analysis, based on a rigorous selection of studies, revealed that while there was a trend towards reduced mortality with restrictive fluid management in septic patients, this difference did not reach statistical significance. Notably, restrictive fluid strategies were associated with a shorter duration of mechanical ventilation, aligning with previous research. Importantly, no concerning signals of adverse effects were identified beyond the initial resuscitation phase. However, the analysis found no significant differences in ICU length of stay, risk of AKI or need for RRT, and hospital length of stay. It is evident that more high-quality studies are needed to establish conclusive findings, but these results contribute to the ongoing discussion on fluid management in sepsis and septic shock, emphasizing the potential benefits of a restrictive approach in specific clinical contexts.
